# Long-Term Effects of Maternal Depression during Postpartum and Early Parenthood Period on Child Socioemotional Development

**DOI:** 10.3390/children10101718

**Published:** 2023-10-23

**Authors:** Anna Suarez, Liubov Shraibman, Vera Yakupova

**Affiliations:** 1 Department of Psychology, Lomonosov Moscow State University, 125009 Moscow, Russia; anna.suarez.fig@msupress.com; 2 Interregional Nonprofit Organization for Advocating for Practical, Informational, and Psychological Support for Families in Pregnancy, Labor & Birth, & Postpartum “Association of Professional Doulas” (INO “APD”), 127253 Moscow, Russia; doula.nsk@gmail.com

**Keywords:** postpartum depression, maternal depression, child emotional development, early parenthood, long-term effects of depression

## Abstract

Most research on the impact of maternal depressive symptoms on child development is conducted during the first year postpartum. Findings on long-term effects of maternal depression are still contradictory and underexplored. The present study investigates the long-term impact of maternal depression during the postpartum and early parenthood periods on child behavioral problems at the mean age of 2.25 years. The Edinburgh postnatal depression scale and Beck depression Inventory were used to assess postpartum and early parenthood depression, respectively. The Child Behavior Checklist was used to examine child’s behavioral problems. The regression analysis showed statistically significant associations between child behavioral problems and maternal depression during early parenthood and no significant associations with postpartum depression. Four maternal depressive symptoms’ trajectories were identified: consistently low, consistently high, decreased, and increased. The children of mothers with consistently high depressive symptoms at both research stages had the most significant total, internalizing, and externalizing problems. Children of mothers whose depressive symptoms decreased over time had the lowest scores in all three domains of behavioral problems. It is extremely important to implement programs for screening and early intervention for maternal mental health problems that could greatly influence the well-being of women and their children’s development.

## 1. Introduction

Mental disorders stand as prominent contributors to the overall burden on global health. According to the Global Burden of Diseases, Injuries, and Risk Factors Study (GBD), depressive and anxiety disorders were the two most incapacitating mental conditions, ranking among the top 25 leading causes of burden worldwide [1]. While depression is prevalent across the lifespan for both sexes globally [2], the World Health Organization (WHO), International Monetary Fund (IMF) and other international organizations emphasize the significance of maternal depressive disorders as an issue of global population health as they are well known to contribute to infant and child morbidity [3,4,5]. Poor outcomes for children may include lower birth weight, suboptimal infant growth, decreased breastfeeding rates, developmental delay, behavioral problems, compromised executive functions and other short- and long-term physical and mental health problems [3,6,7,8].

Maternal depressive symptoms during pregnancy and the first year of postnatal life are highly prevalent, with some studies indicating their levels as being up to 7–20% among pregnant women [9] and as high as 10–26% during the postpartum period [10]. Inconsistencies in the prevalence of postpartum depression (PPD) as well as its reported effects on child development indicate that the chronicity and severity of maternal depressive symptoms are important in determining child outcomes. Therefore, several studies attempted to identify distinct depressive symptoms’ trajectories in order to clarify the risk factors for maternal and child health outcomes [6,9,11]. Fisher et al. [12] distinguished three trajectories of depressive symptoms during the first year of postpartum period in 507 mothers with PPD: gradual remission (50.4%), partial improvement (41.8%) and chronic severe symptoms (7.8%). The other study based on a large British population-based cohort revealed four trajectories, namely resilient (74.3%), improving (9.2%), emergent (4.0%), and chronic (11.5%) [13]. The incidence of persistent or chronic depressive symptoms can reach 2.8–11.5% and these conditions are linked with more severe symptoms in infants and children [6,13].

Heterogeneous trajectories and symptom profiles of PPD and the risk factors for child development were discussed in the recent systematic review by Ahmed Waqas et al. [6]. According to their results, more severe and debilitating trajectories of PPD were associated with poorer cognitive and motor child development, poorer expressive language, more externalizing and internalizing behaviors in both adolescence and adulthood, more emotional problems, poorer peer relationships, higher rates of depression in adolescence, poorer academic performance, higher emotional dysregulation, poorer postpartum bonding, higher risk for chronic illnesses and other health problems in the offspring of different ages [6].

Studies investigating long-term outcomes of maternal depression are rare and usually include small sample sizes. However, there are several research papers that should be highlighted. The study of Cents et al. [14] assessed the association between the intensity of maternal depressive symptoms at 2, 6 and 36 months postpartum and child problem behavior at 3 years. Authors identified four distinct trajectories of maternal depressive symptoms, assessed with the Brief Symptom Inventory—‘no’ (34%), ‘low’ (54%), ‘moderate’ (11%) and ‘high’ (1.5%). The results revealed that children of mothers assigned to higher trajectories had significantly more behavioral problems than children of mothers assigned to lower trajectories [14].

A prospective observational study of 5801 parent–adolescent trios investigated the association between maternal and paternal depression and anxiety measured at 8 weeks, 8 months, 1.5 and 2.5 years postpartum, executive function (EF) at age 8 and academic achievement at the end of compulsory school at age 16 [15]. According to the results, 36% of adolescents of persistently depressed mothers failed to ‘pass’ math at the end of high school compared to 27% of adolescents of nondepressed mothers, with these associations partially explained by the disruption in different components of EF. Interestingly, there was no evidence of an independent association of paternal mental health with impaired child EF or adolescent exams [15].

Similarly, Netsi and colleagues [8] attempted to examine the association between the levels of persistence and severity of PPD and child outcomes, such as behavioral problems at 3.5 years of age, mathematics grades at age 16 years at the end of compulsory school and depression at age 18 based on a substantial sample of over 3600 pairs. According to the results, children of women with persistent and severe depression are at an increased risk for behavioral problems by age 3.5 years as well as lower mathematics grades and depression during young adulthood. Furthermore, women with persistent PPD are also at a higher risk of experiencing significant depressive symptoms 11 years after childbirth.

The Canadian-population-based cohort of 1983 women that additionally included multiple measurements of depressive symptoms during pregnancy identified distinct trajectories of maternal depressive symptoms from mid-pregnancy to one year postpartum (low level (64.7%); early postpartum (10.9%); subclinical (18.8%) and persistent high (5.6%)) and examined the associations between these trajectories and child behavior problems at age 3 [9]. The proportion of children with elevated behavior symptoms (hyperactivity/inattention, physical aggression, and separation anxiety symptoms) were highest in the group where mothers had persistent high depressive symptoms, followed by mothers with moderate symptoms (early postpartum and subclinical trajectories), and were lowest for minimal symptoms.

Finally, Subbiah and colleagues found that higher PPD levels during the first 6 months postpartum were associated with a worse socioemotional development of infants at 12 months of age in a sample of 1843 mother–child dyads [16].

Thus, although convincing evidence suggests that maternal depressive symptoms, specifically their intensity and duration, present risks for their children’s health and development, the exact effects of PPD on the socioemotional child development are still poorly investigated. In Russia, on the contrary, a recent study with a modest sample size (n = 251) that focused on emotional development in preschoolers found no significant association with maternal depression at a child’s mean age of 4.92 [17]. However, although the studies have consistently indicated a high prevalence of PPD ranging between 25% and 46% [18,19,20], to date there have been no longitudinal studies that examined the long-term impact of PPD, maternal depression during the early years and the depressive symptom trajectories on child socioemotional development.

Therefore, the main objective of the present study was to investigate the long-term effects of maternal depression during the early stages of parenthood on child socioemotional development. Namely, the association of maternal depression during the first 12 months postpartum and 12–24 months later with child behavioral and emotional problems was examined. Furthermore, the trajectories of depressive symptoms of the Russian women from the postpartum period to early childhood and their association with child behavioral and emotional problems at the mean age of 2.25 years were explored. It was hypothesized that both PPD and concurrent depression were associated with higher levels of child behavioral problems. It was further expected to see that children of women with consistently high depressive symptoms throughout the early years of parenthood are at a higher risk for internalizing, externalizing and total problems than children of women with consistently low depressive symptoms.

## 2. Materials and Methods

### 2.1. Procedure and Participants

Data for this longitudinal study come from three cohorts of Russian women who gave birth within 12 months prior to the data collection in February–March 2020, February–March 2021, and May–September 2022 (Stage 1). Participants were invited to take part in the web-based survey via social media (e.g., Instagram, Facebook and VK communities for expecting and new parents, perinatal health professionals’ and influencers’ pages), childbirth education classes and communities for new parents as well as from the doctors and midwives in maternity hospitals and healthcare clinics. In total, 4831 women who fulfilled the inclusion criteria (they were 18 years old or above, gave birth within the previous 12 months to live-born children, could read and type in Russian and childbirth took place in Russia) completed the survey in 2020–2022. In August 2022–July 2023, when their children were at the mean age of 2.25 years old (SD = 0.59), the participants received an invitation via email to take part in the next stage of the study and answer the questions about their mental health and their child’s development (Stage 2). If they expressed interest in taking part in the study, they received the printed survey forms via mail. Of all the participants from the three cohorts, 589 returned the filled-in questionnaire forms. After the quality control procedures (matching the unique ID numbers from Stage 1, excluding duplicates, excluding those living outside Russia and excluding those who did not provide critical information such as child sex) 293 mother–child pairs with full information from both stages of the study comprised the sample for this study.

### 2.2. Ethical Considerations

The Ethical Committee of the Russian Psychological Society at Lomonosov Moscow State University approved the present study (No: 345/2019). All participants provided their informed consent using the online tool Testograph prior to filling in the survey. The study was conducted in accordance with the Declaration of Helsinki.

### 2.3. Depression Measures

At Stage 1 mothers completed the Edinburgh Postnatal Depression Scale (EPDS) [21], a validated and commonly used tool to assess pre- and postnatal depressive symptoms. The EPDS consists of ten questions rated on a 4-point Likert scale, ranging from 0 to 3, which indicates how the mother has felt during the previous seven days. A score of 10 and higher is suggested to indicate clinically significant symptoms of depression [21]. The validated Russian version (Cronbach’s α = 0.87) [18] was used, with the reliability in the present study of Cronbach’s α = 0.88.

At Stage 2 women completed the 21-item Beck Depression Inventory (BDI-II) [22] for depressive symptoms over the previous 7 days. The validated Russian version (Cronbach’s α = 0.91) was used [23], with a similar high internal consistency in the present sample (Cronbach’s α = 0.87).

### 2.4. Child Development Measures

At the child’s mean age of 2.25 years (SD = 0.59), mothers completed the Child Behavior Checklist (CBCL 1½—5) on behavioral and emotional problems [24]. The total problems t-score was calculated based on the 99 problem items rated on a scale from 0 (not true) to 2 (very or often true), along with its two subscales, the internalizing and externalizing problems t-scores. The internal validity of the scales in the Russian version [25] were comparable with the one reported in the original study [24] (Cronbach’s α = 0.75 and 0.76, respectively), with a slightly higher value in the present sample (Cronbach’s α = 0.82).

### 2.5. Demographic Characteristics

The survey further included the information on maternal age, level of education (higher/secondary), family status (married/have a partner/single), socioeconomic status (SES) (low/middle/high), place of living (city, small town, countryside) and parity. Furthermore, at Stage 1 women reported obstetric characteristics, such as mode of birth (vaginal/planned cesarean/emergency cesarean/instrumental birth), while at Stage 2 they indicated their child’s age, sex, and chronic medical conditions.

### 2.6. Covariates

Child age (Stage 2), child’s sex, child’s chronic medical conditions, gestational age at birth, mode of birth, maternal age at testing (Stage 2), maternal level of education, family status, SES, place of living and parity were included in the analyses as covariates.

### 2.7. Statistical Analysis

Spearman correlation analysis was used to examine the associations between CBCL scores, maternal age, and the child’s gestational age at birth and between Edinburgh postnatal depression scores and Beck Depression Inventory scores.

The data were normally distributed for all the predictor and outcome variables (*p*-values for all <0.05); thus, the parametric tests were used to explore the associations between them. Namely, univariate analysis was used to explore the association between CBCL scores and a child’s gender and chronic medical conditions. Multiple linear regression analysis examined the association between maternal depression on both stages and child externalizing, internalizing and total problems at toddlerhood. Regression Model 1 was adjusted for child age and sex, Regression Model 2 was further adjusted for the child’s chronic medical conditions, gestational age at birth, mode of birth, maternal age at testing (Stage 2), maternal level of education, family status, SES, place of living and parity.

As child behavioral problem scores are not independent of each other but, rather, represent a hierarchical structure, the associations of maternal PPD and concurrent depression with early childhood total problems were examined first, followed by the examination of the associations with the child’s internalizing and externalizing problems using linear regression analyses. Problem scores were standardized to facilitate interpretation.

The analysis of depressive symptoms’ trajectories was performed with the Wilcoxon Rank Sum Test. The differences between the 4 identified groups were statistically significant (*p* value < 0.05). Each group consists of more than 5% of the total sample.

The data were homogeneous for all the variables (*p*-values for all > 0.15). The level of significance was set to α = 0.05.

All analyses were performed using SPSS 27 software [26].

## 3. Results

The sample was mainly represented by married women with higher education, living in the cities. The characteristics of the sample are presented in Table 1. Of them, 100 (34.1%) women reported clinically significant symptoms of PPD (Stage 1), and 32 (10.9%) participants had clinically significant concurrent depressive symptoms (Stage 2).

There were no significant gender-dependent differences in CBCL scores (*p*-values for all >0.24). However, both total (F = 7.23, df = 292, *p* < 0.01), externalizing (F = 3.88, df = 292, *p* = 0.05) and internalizing (F = 6.94, df = 292, *p* < 0.01) problems were significantly higher in the group of children with chronic medical conditions.

The total scores further negatively correlated with maternal age (rho = −0.12; *p* < 0.05). Internalization problems scores negatively correlated with gestational age at birth (rho = −0.13; *p* < 0.05).

The regression model showed no statistically significant association between PPD and any of the problem scales scores. However, concurrent maternal depression was significantly associated with all three scales when adjusted for child age and sex, and the results remained significant for externalizing and total problems in the fully adjusted model (see Table 2).

### Trajectories of Depressive Symptoms

**Figure 1:**
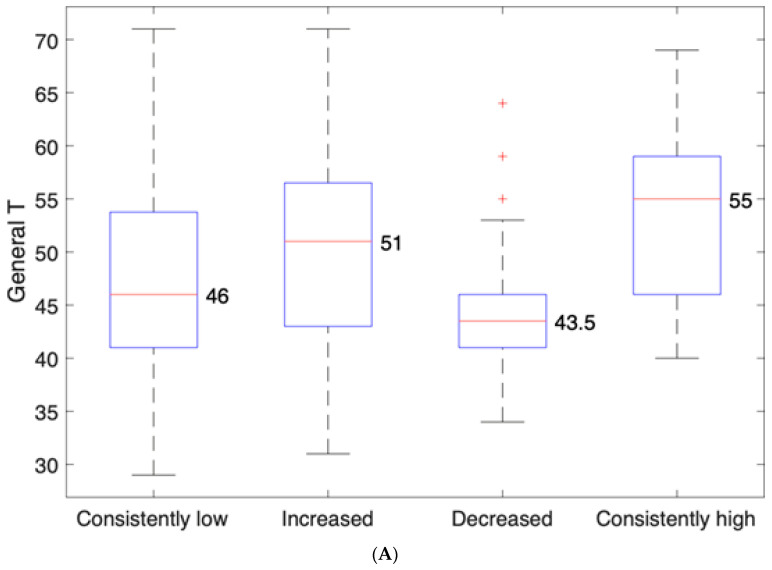

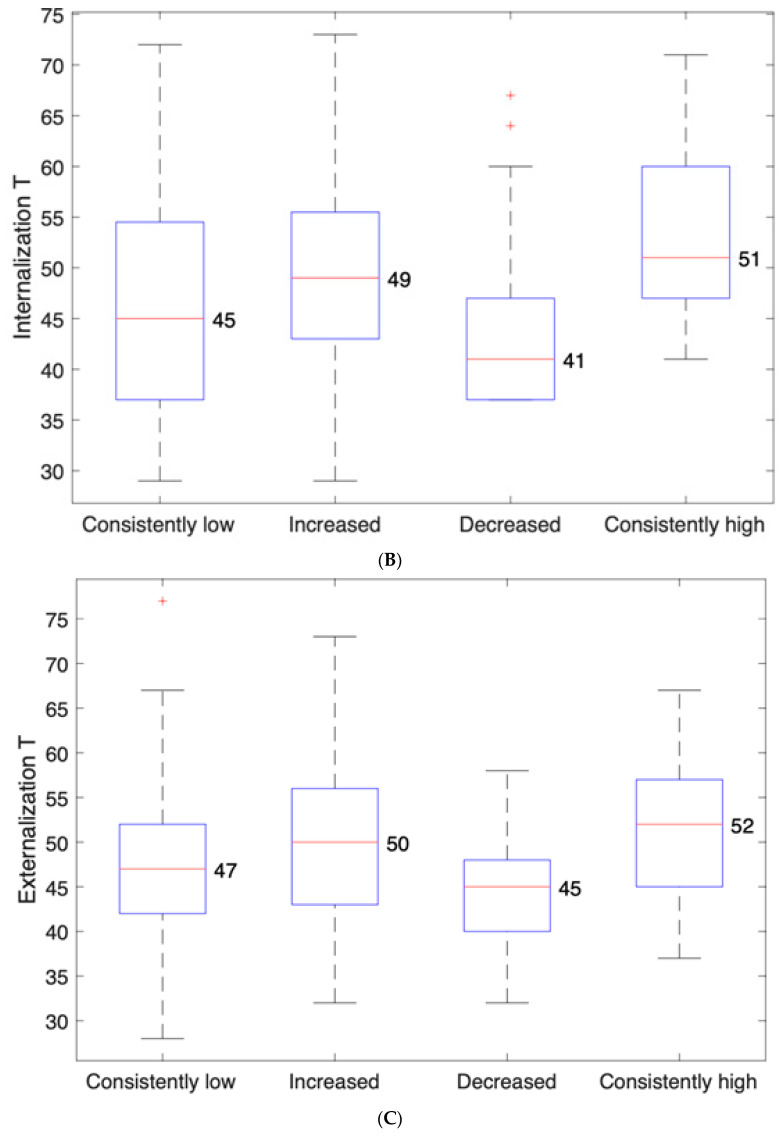
(**A**) Average total CBCL scores for different symptoms trajectories. (**B**) Average internalization problems scores for different symptoms trajectories. (**C**) Average externalization problems scores for different symptoms trajectories.

PPD measured by the EPDS scores (Stage 1) significantly correlated with the BDI scores (Stage 2) (rho = 0.42; *p* < 0.01). Based on the results obtained at the two assessment stages we identified four trajectories of depressive symptoms development: consistently low, consistently high, decreased symptoms (i.e., the symptoms were lower at Stage 2 in comparison to Stage 1) and increased symptoms (i.e., the symptoms were higher at Stage 2 in comparison to Stage 1) (see Table 1). There were statistically significant differences between the consistently low and decreased groups and the consistently high and increased groups in the total problem scores (*p* = 0.003), externalizing problems (*p* = 0.002) and internalizing problems (*p* = 0.002). The strongest differences in all three domains were between the consistently high and decreased groups. Figure 1A shows that children of women with consistently high depressive symptoms from postpartum to early parenthood on average had a t-score = 55 for the total behavioral problems, while children of women whose depressive symptoms decreased with time had a mean t-score = 43.5 for the total behavioral problems (*p* = 0.002). Similarly, Figure 1B shows that the internalization scores were significantly lower in the group of children of the mothers whose symptoms decreased (t-score = 41) in comparison to those whose mothers had consistently high depressive symptoms (t-score = 51) (*p* = 0.016), and the externalization scores were also lowest in the maternal decreased symptoms group (t-score = 45), while the highest externalization problems were among children with consistently high depressive symptoms (t-score = 52) (*p* = 0.022) (Figure 1C).

## 4. Discussion

The aim of this study was to examine the long-term effects of maternal depressive symptoms during postpartum and early parenthood periods on child socioemotional development. According to the results, child behavioral problems were associated with maternal depressive symptoms concurrent with the child assessment period, but not with PPD symptoms, and these results remained significant in the presence of important covariates (child’s chronic medical conditions, gestational age at birth, mode of birth, maternal age at testing (Stage 2), maternal level of education, family status, SES, place of living and parity). Furthermore, the obtained results indicate that children of mothers with consistently high symptoms of depression had significantly more behavioral problems measured with CBCL (1½—5) in comparison to children of mothers with other trajectories of depressive symptoms. Finally, our findings show that children’s behavioral problems scores were elevated in those children whose mothers did not have depressive symptoms at Stage 1 of the study but had high depressive symptoms at Stage 2 and the behavioral problems in all three domains were the lowest in the group of children whose mothers’ depressive symptoms decreased over time.

Contrary to our hypothesis, child behavioral problems were significantly associated with concurrent depression, but not the PPD. These results seem to contradict the systematic review of the consequences of maternal PPD for the infant outcomes [27] as well as several previous studies that showed the association of elevated PPD symptoms during the first months of the child’s life with child total, internalizing and externalizing problems at 8 years [28], low social competence at the age of 8–9 years [29] and low social competence and externalizing problems at age 16–17, but only in boys [30]. However, at a closer look our findings follow the same trend as the work of Closa-Monasterolo and colleagues, where although maternal PPD was significantly associated with child behavioral problems in the regression analysis, the effect size was the lowest compared to the association with concurrent depression and PPD with concurrent depression together [28]. Furthermore, when the authors compared the children of mothers with PPD only and children of mothers without depression at either point, they found no statistically significant differences, in contrast to mothers with concurrent only and PPD with concurrent depression [28]. Similar to our findings, a small study from Iran Abdollahi and colleagues showed that after the adjustment for covariates, children of women with current depression and both PPD and current depression had more developmental disabilities, while there was no such association for women with PPD only [31].

This contradictory evidence might be related to the differences in the measurement tools used to assess PPD. As there was no access to any objective clinical information for diagnosing PPD, we opted to utilize the frequently employed EPDS score threshold of 10 or above [21] to gauge the prevalence of PPD within our study cohorts at Stage 1. Nevertheless, it is worth noting that this particular threshold may be overly sensitive, potentially leading to an overestimation of the PPD rate [32,33], which, in turn, might have prevented us from detecting the consequences of more severe PPD symptoms on child development. Consequently, additional research employing alternative diagnostic instruments or EPDS thresholds aligned with both DSM-5 and ICD-10 criteria for depression is imperative. On the other hand, this might also indicate that maternal and child experiences throughout the time following the postpartum period are crucial for understanding the risk factors for child development; thus, the inclusion of the important covariates such as maternal current age, family status, education, SES and child chronic illnesses among others that were included in our study, may be crucial when exploring the true long-term effects of PPD.

The trajectories analysis among Russian women is in line with the previous body of literature from other countries indicating that children of mothers with persistently high depressive symptoms are at an elevated risk for developmental difficulties [8,15]. There were four distinct trajectories: women with consistently low depressive symptoms, those whose symptoms decreased over time, those whose symptoms increased over time and women with consistently high depressive symptoms. These trajectories mirror those found in the Avon Longitudinal Study of Parents and Children (ALSPAC) study, which capitalized on the cohort of 14,170 mothers who filled in the EPDS 10 times from 18 weeks of pregnancy up to 134 months postpartum, and the four distinct trajectories these authors found were minimal symptoms, increasing symptoms, persistent symptoms and decreasing symptoms [34].

Intriguingly, we found that while the highest total, internalizing and externalizing scores were among the children of women with consistently high depressive symptoms, the lowest scores in all three domains were in the group of children whose mothers had lower depressive symptoms at Stage 2 in comparison with Stage 1, even in comparison with the consistently low group. Correspondingly, Park et al. showed that children whose mothers became less depressed over time had lower levels of behavioral problems at age 3, executive functions and internalizing and externalizing scores at age 6, and fewer reported attention deficit/hyperactivity disorder (ADHD) symptoms at age 6 in comparison to those whose mothers remained less depressed over time, thus suggesting that if maternal depressive symptoms improve over the first three years postpartum, their children’s outlook may be comparable or even more favorable to those whose mothers had consistently low depressive symptoms [35].

As Mughal and colleagues showed that the strongest predictors of the persistent symptom trajectory included a maternal history of depression and inadequate social support [34], the same mechanisms might affect the children as they share the environment with their mothers. It has been widely demonstrated that a history of trauma and abuse, childhood maltreatment, isolation, conflict with intimate partners, poverty and other social factors present risk both for depression in adults and for developmental delays and behavioral problems in children [36,37,38,39,40]. Therefore, the focus of the future studies as well as intervention and prevention programs should address not only maternal depression and its effects on child development, but rather the improvement of social support both for the mother and her children.

Moreover, our findings confirm the value of early interventions, as mothers who suffered from depression during the postpartum period but had fewer depressive symptoms two years later had children with the lowest scores on all three domains of behavioral problems. It is possible that women in this group were provided adequate treatment with psychotherapy or/and medication as well as received support from their family and other social groups, thus empowering them as mothers and developing useful skills for coping with the stress of parenthood.

Finally, the depressive symptoms might have decreased over time as the PPD was rather a reactive form of depression in that group of women in contrast to the consistently high symptoms group who may have an endogenous form of depression that might be more severe and prolonged [41]. In the latter, women might be genetically more vulnerable to depression and, therefore, their children might also be genetically more vulnerable for psychopathology [42,43], which is reflected in their behavioral problems early in life.

Therefore, taken together, our findings suggest that interventions as early as during pregnancy and early months postpartum are of paramount importance to mitigate the effects of maternal emotional discomfort on child development, and improve the health outcomes for both the mother and her children. Maternal depression is a modifiable risk factor for child socioemotional and behavioral problems that can be addressed with psychotherapy, pharmacological therapy, and adequate social support. According to the costs of perinatal mental health problems report by the London School of Economy, in the UK alone the known costs of perinatal mental health problems per year’s birth exceed 8 billion pounds; of them, only 28% relate to the mother, while 72% relate to the child’s subsequent health problems [44]. Thus, it is essential to develop and implement effective prevention, screening and early intervention programs that could have a significant impact on public health, the economy and quality of life of individual families.

## 5. Conclusions

Maternal depression is a serious public health problem that presents risk for socioemotional development of their children. The objective of this study was to investigate the long-term impact of maternal depression during the postpartum and early parenthood periods on child behavioral problems at the mean age of 2.25 years. Our results do not support the previous evidence of long-term consequences of PPD during the first 12 months postpartum alone on child total, internalizing and externalizing problems at age 2, but emphasizes the effects of the concurrent maternal depressive symptoms on child behavioral problems. Furthermore, our findings highlight that children of mothers with consistently high depressive symptoms from the postpartum to early parenthood period have the most significant total, internalizing and externalizing problems. However, there is also a hopeful discovery showing that children have the lowest scores in all three domains of behavioral problems if maternal depressive symptoms decreased over time. Therefore, it is imperative to formulate and implement programs for prevention, screening and early intervention for maternal mental health problems that could greatly influence the well-being of women and their children, as well as the public health sector and global economy.

## 6. Strengths and Limitations

The strengths of our study include the longitudinal study design, which allows us to estimate long-term effects of maternal depression on child development. The data on PPD were collected during the postpartum period, which increases its reliability. The use of validated questionnaires and controls for important covariates (such as child’s gestational age at birth, chronic medical conditions, maternal education, and socioeconomic status) can also be considered as strengths.

However, several limitations should be noted.

The data were mostly collected only in big cities among women with higher education and the future investigation of other social strata is required.

The information on child development is based only on the mother’s reports, which could have been biased by the presence of depressive symptoms. The inclusion of reports from fathers and other caregivers could enrich the obtained results and increase their reliability. Our findings also lack objective medical information on maternal depression and child medical conditions and rely solely on self-reports, which is a common limitation in the countries without available registry-based data.

## Figures and Tables

**Table 1:** Characteristics of the sample.

	All	Consistently Low Depressive Symptoms	Consistently High Depressive Symptoms	Decreased Depressive Symptoms
N	293	139	44	22
Week of gestation at birth (M(SD))	39.3 (1.95)	39.5 (1.5)	38.9 (2.5)	39.4 (1.9)
Mode of birth (N (%))				
Vaginal	196 (66.9%)	102 (73.4%)	21 (47.7%)	10 (45.5%)
Instrumental birth	10 (3.4%)	6 (4.3%)	0 (0%)	0 (0%)
Planned CS	37 (12.6%)	7 (5%)	14 (31.8%)	4 (18.2%)
Emergency CS	50 (17.1%)	24 (17.3%)	9 (20.5%)	8 (36.4%)
Mothers’ age (M (SD)	33.4 (4.3)	33.5 (4.3)	32.6 (4.2)	33.7 (3.8)
Education (N (%))				
Higher	279 (95.2%)	136 (97.8%)	39 (88.6%)	21 (95.5%)
General Secondary	14 (4.8%)	3 (2.2%)	5 (11.4)	1 (4.5%)
SES (N (%)				
low income	41 (14.0%)	15 (10.8%)	8 (18.2%)	1 (4.5%)
middle income	176 (60.1%)	80 (57.6%)	28 (63.6%)	15 (68.2%)
high income	76 (25.9%)	44 (31.7%)	8 (18.2%)	6 (27.3%)
Marital status (N (%))				
Married	267 (91.1%)	128 (92.1%)	39 (88.6%)	20 (90.9%)
Have a partner	12 (4.1%)	4 (2.9%)	4 (9.1%)	0 (0%)
Single	14 (4.4%)	7 (5%)	1 (2.3%)	2 (9.1%)
Place of living				
city	270 (92.2%)	130 (93.5%)	39 (88.6%)	21 (95.5%)
town	6 (2%)	3 (2.2%)	2 (4.5%)	1 (4.5%)
countryside	17 (5.8%)	6 (4.3%)	3 (6.8%)	0 (0%)
Parity (N (%))				
1	176 (60.1%)	86 (61.9%)	28 (63.6%)	10 (45.5%)
2	62 (21.2%)	25 (18%)	13 (29.5%)	7 (31.8%)
>2	55 (18.7%)	28 (20.1%)	3 (6.9%)	5 (22.7)
Child’s chronic medical conditions (N (%))				
None	228 (77.8%)	114 (82%)	30 (68.2%)	15 (68.2%)
At least one	65 (22.2%)	25 (18%)	14 (31.8%)	7 (31.8%)
Child gender (N (%))				
boy	130 (44.4%)	62 (44.6%)	21 (47.7%)	11 (50%)
girl	163 (55.6%)	77 (55.4%)	23 (52.3%)	11 (50%)
Child’s age at stage 2 (M(SD))	2.25 (0.59)	2.3 (0.6)	2.2 (0.6)	2.3 (0.6)
Edinburgh depression scale score (M(SD)	9.3 (6.2)	5.9 (3.6)	18.7 (5.5)	17.5 (3.3)
Beck depression Scale score (M(SD)	9.3 (7.9)	3.8 (2.7)	18.5 (8.5)	4.4 (2.4)
Internalizing child problems (M(SD)	47.8 (9.7)	46.2 (9.9)	52.9 (8.7)	44.8 (8.9)
Externalizing child problems (M(SD)	48.5 (8.7)	47.1 (8.7)	51.8 (7.7)	45.2 (6.2)
Total CBCL score (M(SD)	48.9 (8.9)	47.3 (9.1)	53.5 (7.5)	45.2 (7.2)

**Table 2:** Regression models: association between depression at Stage 1 and Stage 2 and CBCL scores (Stage 2).

	Stage 1 (Postpartum)		Stage 2 (Early Childhood)	
Model 1	β (95% CI)	*p*-value	β (95% CI)	*p*-value
Internalizing problems	0.115 (−0.008; 0.24)	0.067	0.162 (0.03; 0.28)	0.01
Externalizing problems	0.057 (−0.06; 0.18)	0.37	0.195 (0.07; 0.31)	0.002
Total score	0.082 (−0.04; 0.21)	0.19	0.215 (0.09; 0.33)	0.001
Model 2	β (95% CI)	*p*-value	β (95% CI)	*p*-value
Internalizing problems	0.13 (−0.07; 0.33)	0.19	0.14 (−0.02; 0.30)	0.083
Externalizing problems	−0.003 (−0.18; 0.17)	0.97	0.16 (0.01; 0.30)	0.031
Total score	0.06 (−0.12; 0.24)	0.53	0.16 (0.02; 0.31)	0.025

Model 1 is adjusted for child age and sex, Model 2 is further adjusted for child’s chronic medical conditions, gestational age at birth, mode of birth, maternal age at testing (Stage 2), maternal level of education, family status, SES, place of living and parity. Significant *p*-values are bolded (*p* < 0.05). β refers to standardized regression coefficients from a multiple linear regression model. The 95% CI refers to the 95% confidence interval.

## Data Availability

The anonymized dataset, syntaxes, and the survey form (in Russian) may be provided upon a reasonable request.
